# Cognitive behaviour therapy-informed guidelines for type 2 diabetes self-management: A Delphi method study

**DOI:** 10.1177/13591053261428853

**Published:** 2026-03-23

**Authors:** Elné Visagie, Elmari Deacon, Rumando Kok

**Affiliations:** 1 University of Pretoria, Faculty of Humanities, South Africa; 2 North-West University, Potchefstroom, South Africa

**Keywords:** cognitive behaviour therapy, CBT-informed guidelines, type 2 diabetes, self-management, the Delphi method

## Abstract

This study aimed to evaluate the content and quality of cognitive-behavioural therapy-informed guidelines that healthcare professionals can implement to improve self-management of adults with type 2 diabetes. A qualitative design using the Delphi method was employed. This method facilitated two rounds of feedback from a panel of 12 healthcare professionals, enabling collaborative refinement of the guidelines. Four major themes emerged during the first round of feedback: (1) the CBT-informed guidelines are clear and concise, (2) a need for information regarding role clarification, (3) the need to consider the patient’s readiness to change and (4) the consideration that the guidelines may be time-consuming. The second round reiterated the clear, practical nature of the guidelines. The guidelines propose a framework that supports a holistic approach to self-management. The guidelines’ flexibility enables healthcare professionals to adapt them to the diverse needs of individuals with type 2 diabetes.

## Introduction

The rise of type 2 diabetes mellitus (T2DM) has become a global epidemic with a noticeable prevalence in developing countries (Koochaksaraee et al., 2022). The epidemiology of the chronic condition is complex, as it is an amalgamation of biological factors, lifestyle choices and preferences, and socioeconomic factors (Geneti et al., 2022). The interaction of these factors contributes to the severity of the individual’s condition. It can cause acute and long-term diabetes-related complications, including cardiac complications, retinopathy, chronic kidney disease, and neuropathy (Vlachou et al., 2022). In addition to its comorbidities and complications, the condition entails a complex behavioural aspect, as it requires intentional actions by the individual to maintain health and well-being (Cornely et al., 2022). T2DM management is based on self-management, with the individual as the primary decision-maker responsible for more than 95% of specific activities required to achieve management goals (Koochaksaraee et al., 2022; Pienaar and Reid, 2020). Self-management levels vary, with adherence to medication and self-management ranging from 36% to 93% (Murwanashyaka et al., 2022). Individuals’ complex inner dynamics, characteristics, and lived experiences collectively shape perceptions and beliefs about T2DM, which can hinder or facilitate self-management practices (Nantha et al., 2019). Hence, self-management poses a formidable challenge, as some individuals may lack awareness of how these internal dynamics affect their self-management practices or struggle to confront and alter obstructive thoughts, emotions, and behaviours (Fiqri et al., 2022). Effective self-management occurs when individuals align their resources, principles, routines, and preferences with a nutritious diet, increased physical activity, blood glucose monitoring, medication adherence, prevention, and psychological well-being (Pamungkas et al., 2019). It is a dynamic process of adapting one’s cognitive, behavioural, and emotional responses to the condition to uphold a satisfactory quality of life (Pienaar and Reid, 2020). Numerous studies have indicated that emphasis should be placed on the psychological components influencing self-management (Azami et al., 2018). Cognitive behavioural therapy (CBT) is a psychological approach that addresses thoughts and behaviours that impede an individual’s quality of life and, in the case of T2DM, those that impede effective self-management (Koochaksaraee et al., 2022). CBT provides a framework for understanding innate self-management beliefs by first identifying the salient determinants of emotional responses and external behavioural practices, and then creating conscious connections between these interactions and self-management efficacy through the implementation of more helpful thoughts and behaviours (Nantha et al., 2019). The collaborative nature of CBT and the patient involvement required by self-management have proven complementary in facilitating effective self-management practices and glycaemic control (Clarke et al., 2019; Pan et al., 2020; Visagie et al., 2023). The goals of CBT for health-related conditions include empowering the patient, promoting treatment adherence, cultivating responsibility, and facilitating self-efficacy by implementing cognitive restructuring and cognitive disengagement, promoting self-and disease evaluation, and employing continuous action plans to maintain effective self-management practices (Cornely et al., 2022). It enables individuals to recognise that they have control over their self-management and empowers them to become active participants in improving it by employing decision-making, resource utilisation, and problem-solving skills (Motevalli et al., 2023). There is a need to conduct research related to CBT interventions and self-management that addresses core self-management-related thoughts, behaviours, and emotions and does not primarily focus on how CBT can improve the pathology that coincides with or is developed by the diagnosis and condition (e.g. depression; Koochaksaraee et al., 2022; Visagie et al., 2025). A further consideration is the context in which T2DM diagnosis is increasing. Mutyambizi et al. (2020) reported that 80% of individuals diagnosed with diabetes reside in low and middle-income countries, and there is a need for interventions that are cost-effective, accessible, and time-sensitive. Similarly, Pienaar and Reid (2020) found that, within these settings, there is a need for improved community awareness of diabetes, face-to-face information sharing, and training platforms for healthcare providers (HCPs) who consult with individuals diagnosed with T2DM. The advantage of CBT is that its structure and approach align with these requirements. It amalgamates the individual’s internal dynamics with practical skills, making it more user-friendly for HCPs with limited time and psychological training. Therefore, this study presented CBT-informed guidelines for promoting effective self-management practices to a panel of experts via the Delphi method. The aim was to evaluate the content, quality, and structure of the CBT-informed guidelines to optimise its usage in healthcare settings. In the future, these guidelines can be presented to HCPs to help create a more extensive network of professionals who can facilitate patients’ understanding of self-management-related cognitions, emotions, and behaviours, and to increase access to resources for individuals with type 2 diabetes who struggle with self-management.

## Methods

### Research paradigm

This study employed an exploratory qualitative research design, utilising the Delphi method to inductively derive feedback on the structure, content, and quality of the CBT-informed guidelines for improved self-management practices (Gossler et al., 2019). The principles of the Delphi method aligned with the study’s aim of obtaining on-the-ground feedback from experts knowledgeable about the demographics of patients in a developing country, the time constraints experienced across different healthcare settings, and the level of resource accessibility (Creswell, 2014). The process enabled the researchers to capture rich, tacit feedback, opinions, and ideas to analyse the potential use of the guidelines in the healthcare context.

### The Delphi procedure

The study employed a two-round Delphi method. The decision to conduct two rounds was based on the study’s primary aim of refining the guidelines through expert feedback, rather than on achieving statistical consensus on numerous predetermined items. This is a widespread approach in health research, as the central goal is to gradually develop and refine an intervention or guideline rather than focus on strict agreement across many points (Niederberger and Spranger, 2020). The first round aimed to gather detailed, open-ended feedback to identify key areas for improvement. The second round was important for presenting the updated guidelines back to the panel. This allowed the experts to check whether their feedback had been incorporated and to suggest final, practical changes. This two-round approach achieved balanced improvement and efficiency, while minimising participant burden (Keeney et al., 2011).

The goal of this study was to obtain a consensus of at least 80% among experts on the evaluation of the guidelines’ content, structure, and quality. This criterion is recommended in health research for balancing methodological rigour with practical feasibility (Diamond et al., 2014; Niederberger and Spranger, 2020; von der Gracht, 2012). Following the first round, reflexive thematic analysis revealed that over 80% of the panel’s feedback converged on four main themes, indicating consensus on the key areas requiring revision. Therefore, the focus of the second round shifted from building consensus to confirming that the revisions made were appropriate and representative.

### Participant recruitment

Experts for the Delphi method were recruited by employing purposive sampling. The expert panel was recruited using specific inclusion criteria established prior to recruitment, in accordance with the Delphi method guidelines (Jünger et al., 2017; Niederberger and Spranger, 2020). Each criterion was designed to elicit the necessary expertise to assess the CBT-informed guidelines. The inclusion criteria were as follows:

1. Experts had to have advanced knowledge and sufficient experience in type 2 diabetes and/or CBT. This was to ensure that the experts could provide feedback that is clinically relevant and theoretically grounded, offering a comprehensive perspective (Foth et al., 2016; Skivington et al., 2021).
2. Registration with a professional regulatory board was required to ensure credibility in their input (Diamond et al., 2014; Jünger et al., 2017).
3. The experts had to have two or more years of professional experience in CBT and/or working with individuals with T2DM. This requirement ensured that the experts had moved past basic training to develop practical expertise relevant to implementation feasibility (Boulkedid et al., 2011; Keeney et al., 2011).
4. They had to have sufficient time to partake in the process. This was to ensure the integrity of the process and reduce attrition bias (Hohmann et al., 2023; Niederberger and Spranger, 2020).

The target panel size of 10–15 experts was determined based on methodological recommendations for heterogeneous Delphi panels. The focus was on gaining insights from different HCPs rather than achieving statistical representativeness (Diamond et al., 2014; Niederberger and Spranger, 2020). The panel composition included general practitioners, diabetes educators/nurses, dietitians, and psychologists to represent the core frontline providers in South Africa’s primary care pathway for T2DM. This ensured that the guidelines were grounded in the practical realities of resource-constrained settings, where specialist access is often limited, and provided complementary expertise across biomedical, educational, nutritional, and behavioural domains that are essential for holistic self-management (Foth et al., 2016). The recruitment process followed a structured path. Initial experts were identified from professional networks within South African healthcare, with a focus on diversity across professions and practice settings. Participant recruitment concluded when a diverse representation was achieved. The panel demonstrated diversity across professions, years of experience, age, and gender. Further considerations for conducting recruitment included that the panel size matched the recommended range (10–15) and that additional recruitment was not expected to yield new perspectives beyond those offered by the current panel. Participants who indicated their willingness to participate received an email containing the informed consent form, an information leaflet explaining the Delphi method, and additional information about the study’s purpose and the expected duration of the two research rounds. The independent person set up a time for an online consent session. Once consent was obtained, the CBT-informed guidelines were emailed to the expert. The panel included 12 experts: 3 psychologists (2 counselling and 1 clinical), 4 dieticians, 3 medical doctors, and 2 registered nurses (diabetes educators). Most panel members were knowledgeable about type 2 diabetes, but not all had experience or exposure to CBT. Table 1 summarises the demographics of the experts who participated in the first and second rounds.

**Table 1.** Demographics of the Delphi panel.

Expert code	Gender	Age	Ethnicity	Profession, years of practice and regulatory board
E1	Female	43	Caucasian	A counselling psychologist with 6 years of experience employing CBT and registered with the HPCSA.^a^
E2	Female	31	Caucasian	A dietician with 8 years of experience working with T2DM, registered with ADSA.^b^
E3	Female	50	Caucasian	A dietician with 26 years of experience working with T2DM, registered with the HPCSA.^a^
E4	Female	37	Caucasian	A medical doctor with 10 years of experience working with T2DM, registered with the HPCSA.^a^
E5	Female	51	Caucasian	A nurse with 26 years of experience working with T2DM, registered with the SANC.^c^
E6	Female	36	Caucasian	A clinical psychologist with 13 years of experience employing CBT, registered with the HPCSA.^a^
E7	Female	60	African	A dietician with 8 years of experience working with T2DM, registered with the HPCSA^a^.
E8	Male	56	Caucasian	A specialised physician with 23 years of experience working with T2DM, registered with the HPCSA.^a^
E9	Female	70	African	A nurse with 17 years of experience working with T2DM, registered with the SANC.^c^
E10	Male	65	Caucasian	A medical doctor with 40 years of experience working with T2DM, registered with the HPCSA.^a^
E11	Female	30	Caucasian	A dietician with 6 years of experience working with T2DM, registered with ADSA^b^ and the HPCSA.^a^
E12	Male	32	Caucasian	A counselling psychologist with 4 years of experience employing CBT and registered with the HPCSA.^a^

a The Health Professions Council of South Africa.

b The Association for Dietetics in South Africa.

c The South African Nursing Council.

### Data collection

The two rounds of Delphi took place between June and September 2023. During the first round, the panel of experts were sent the CBT-informed guidelines manual (which included an explanation of the guidelines), an infographic summarising the CBT-informed guidelines process, and an open-ended questionnaire. The CBT-based guidelines were developed by using previous research conducted by the researcher. This included a rapid review to synthesise the current literature on CBT and self-management (Visagie et al., 2023) and an exploration of the thoughts, emotions, and behaviours of adults with type 2 diabetes through semi-structured interviews (Visagie et al., 2024). The experts could make comments and suggest changes to the manual, answer the open-ended questionnaire, and had a 2-week timeframe to provide their feedback. The first-round questionnaire encouraged the experts to express their overall impressions of the guidelines, identify the strengths and weaknesses, and rate the proposed guidelines out of 100. Lastly, the experts were invited to share any ideas or suggestions to enhance the guidelines’ effectiveness. This round aimed to refine the CBT-informed guidelines by delineating experts’ differing opinions through the identification of themes and consensus (Cataldi and Sena, 2021). The synthesis of responses received in round one, a refined open-ended questionnaire and the refined guidelines were emailed to the experts for the second-round review. Similarly, the experts had 2 weeks to provide their feedback. The second-round questionnaire included two key inquiries. It asked the experts to share their overall impressions of the revised guidelines and to provide feedback on any additional changes that should be incorporated. As defined in the Delphi procedure, this study aimed for an 80% consensus threshold. This consensus was reached after the first round; however, a second round was initiated to address expert feedback and suggestions (Figure 1).

**Figure 1.**
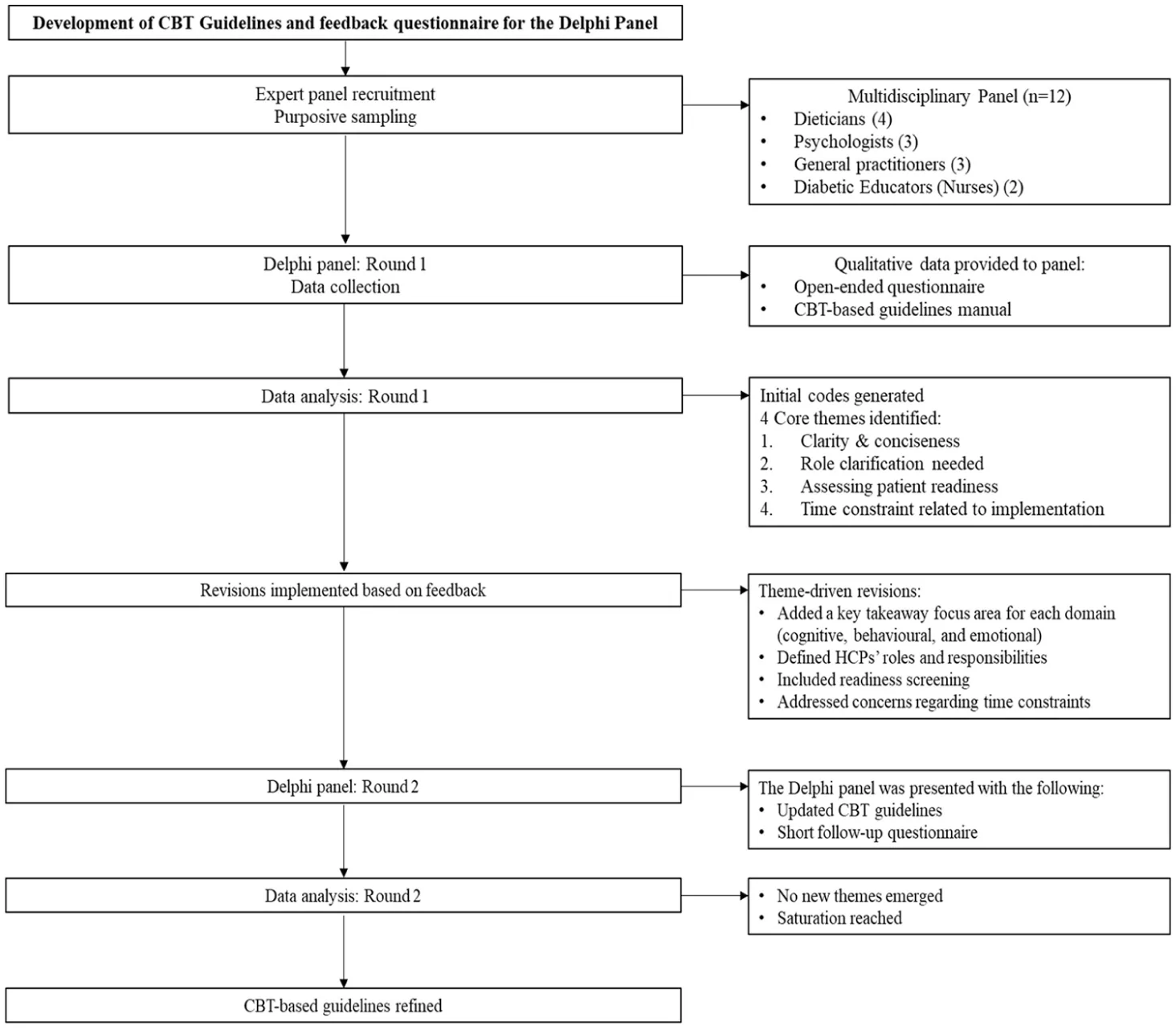
Flow diagram of the Delphi process.

### Data analysis

Data were analysed after each round through reflexive thematic analysis (Braun and Clarke, 2021; Byrne, 2022). The process was inductive and followed the six steps as outlined by Braun and Clarke (2021). The steps included: data familiarisation; identifying codes for the dataset; constructing initial themes; reviewing the initial themes; refining and defining the final themes; and reporting the results (Byrne, 2022). The themes from the Delphi rounds evolved iteratively. During round 1, detailed, qualitative feedback was obtained, comprising questionnaire responses and in-document comments on the draft guidelines. The initial coding stage identified distinct codes, such as practical steps, HCP role confusion, patient motivation, and time pressure. These codes were grouped based on shared meaning and conceptual relationships. Four main themes were developed based on the codes. A co-coder conducted independent data analysis and confirmed that the findings accurately represented the dataset and were interpreted appropriately. This enhanced the accuracy and the credibility of the findings. Furthermore, 80% consensus was achieved among the expert panel, informing revisions. The second round served as a member-check and refinement phase. The analysis of Round 2 feedback did not generate new themes. It confirmed that the initial concerns were sufficiently addressed. The discussion shifted from identifying problems to supporting solutions and proposing minor technical improvements.

### Ethical considerations

The researcher obtained ethical clearance from the Health and Research Ethics Committee (HREC) at the NWU Potchefstroom campus under ethical number NWU-00301-21-A1. Written informed consent was secured from all participants, and their right to withdraw from the study was reiterated. The independent person and informed consent document detailed explanations regarding data storage and handling protocols to ensure transparency. All interview transcripts were coded to safeguard anonymity, ensuring participants’ identities remained confidential in subsequent data dissemination. The experts were assured that they could freely provide constructive feedback and opinions, and as much or as little information as they felt comfortable. The study ensured trustworthiness by adhering to the principles of credibility, dependability, confirmability, transferability, and authenticity (Lincoln and Guba, 1985). The researchers were adequately trained in data collection and analysis methods. Furthermore, reflexivity and member checking ensured the conscientious consideration of subjective predispositions and validated the feedback received during the Delphi rounds.

## Results

Four main themes were identified during round one, namely, (1) the CBT-informed guidelines are clear and concise, (2) there is a need for information regarding role clarification, (3) it is important to consider the patient’s readiness to change before and during the implementation of the guidelines, and (4) the healthcare practitioners anticipate the guidelines to be time-consuming. The second round reiterated the guidelines’ clear and practical nature and suggested technical changes.

### First-round feedback

#### Theme 1: The CBT-informed guidelines are clear and concise

Most of the Delphi panel agreed that the CBT guidelines were clear and concise. The content was informative and well-structured, and highlighted the practical nature of the guidelines. E1 commented, *‘It is precise, clear, and almost a step-by-step guide*’. The examples provided facilitated understanding of the thoughts, emotions, and behaviours experienced by adults with type 2 diabetes. E5 remarked, ‘The content is well explained, and I found the examples to aid *my understanding*’. It enhanced the practicality and user-friendliness of the guidelines by presenting strategies to facilitate patient understanding, participation, and adherence. E2 stated, ‘*The CBT-informed guidelines provide clear and actionable guidance to healthcare professionals. They offer step-by-step instructions, specific interventions, and strategies that can be easily understood and implemented in real-world clinical settings*’. Several experts noted that these guidelines recognise that diabetes management goes beyond medical intervention and incorporates psychological principles that address the emotional and behavioural factors that impact diabetes self-management. ‘*They focus holistically on the patient. It would assist the patient to take ownership of their condition*’ (E4).

#### Theme 2: There is a need for information regarding role clarification

The Delphi panel commented that role clarification was needed regarding which HCP will implement the guidelines, the training required for these individuals, and the HCP’s and the patient’s responsibilities, respectively. E3 stated, ‘*I do touch on individual areas like reframing and Socratic questioning, but employing the whole model is almost impossible. They might ask why they are getting* ‘*emotional counselling’ from a dietician!*’. Additionally, it was suggested that the patient’s expectations and responsibilities should be clearly outlined. E8 summarised that the guidelines should ‘*Contract a patient to participate in CBT for diabetes care and what the expected duration and input from the patient will be’*. The panel indicated uncertainty about which HCPs are the target audience, requiring further elaboration on which HCPs will implement these guidelines and in which settings this will occur. E8 stated, ‘*It is not clear who the audience of the guideline is. HCPs are not defined*’. Furthermore, some panel members highlighted the need to familiarise themselves with the components of CBT. Several panellists noted that individuals may become overwhelmed if not accustomed to CBT, particularly during the working phase of the guidelines. E5 explained, ‘*For someone who is not a psychologist, it gets overwhelming in the working phase’.* E11 stated, ‘*HCPs, other than psychologists, might find it difficult to assist the patient in finding the link between triggers, thoughts, behaviours, and emotions’.* Thus, most panel members indicated that some level of training would be beneficial to enhance the competence and understanding of those implementing the guidelines. ‘*It may require significant training and education for healthcare providers to adopt and implement these guidelines effectively’* (E2).

#### Theme 3: It is important to consider the patient’s readiness to change before and during the implementation of the guidelines

The Delphi panel agreed that the CBT guidelines emphasise a patient-centred approach that promotes collaboration between the HCP and the patient, ensuring that the techniques are tailored to the individual’s circumstances and priorities. E12 commented, ‘*These guidelines allow for and encourage interactive collaboration throughout’.* However, the cornerstone of effective self-management is the patient’s willingness to engage in change, and some experts commented that the guidelines do not sufficiently address this. E2 stated, ‘The *CBT-informed guidelines often assume a certain level of patient motivation and active engagement in the treatment process. However, not all individuals may be ready or willing to participate fully in psychological interventions. Factors such as low motivation, lack of insight, or cognitive impairments may sometimes limit the effectiveness of CBT-based approaches’*. Goal setting was highlighted as a potential strategy to facilitate patient buy-in and motivation. ‘*The goals and aims listed here are correct, but they are still seen as ‘hard work’ or ‘grudge purchases’ because it does not translate into practical benefits to the patient’* (E3). E9 elucidated, ‘*It is important that they agree to the suggestion made, for there will be nothing done about them without them (clearly defined goals)’.* Additionally, experts noted that HCPs should be aware of the patient’s socioeconomic and cultural context factors, which affect goal setting, resource identification, and homework challenges. E9 highlighted, *‘Modifying behaviour may sometimes be challenging because some of our target members may not have choices. Financial or food choices’.* The guidelines emphasised patient empowerment and outlined a process for identifying resources for both HCPs and patients to navigate available options within the patient’s context. E12 remarked, ‘*Some patients may require more individualised intervention catered to their specific needs*’.

#### Theme 4: The healthcare practitioners anticipate the informed guidelines to be time-consuming

A prominent theme that emerged concerned the time constraints faced by HCP. Most of the experts indicated that the implementation of the CBT-informed guidelines would be time-consuming, and was uncertain how this could be integrated into the current interaction time with patients; E4 and E3 explained that ‘D*ue to time constraints, most medical doctors would not implement this’*, and ‘*Dieticians are supported by medical aids for only 2 x 1-hour sessions. The result is that the limited time precludes the dietician from utilising these guidelines’*. Some of the panel mentioned that patient-related factors and HCP training could play a role in initially implementing the guidelines. E7 commented, ‘*I think more time will be spent, especially initially, compared with the usual consultation time because patients are not accustomed to CBT’.* Furthermore, within the public health sector, the patient is not guaranteed to consult with the same HCP each time they visit the healthcare facility. Certain panel members suggested setting an approximate time allocation for each phase of the guidelines, which would facilitate the HCP’s tracking of time spent implementing the guidelines during a consultation.

### Second round feedback

Before the second round commenced, the following theme-based revisions were made to the guidelines, as shown in Table 2, Round 1 Themes and Corresponding Revisions. The refined guidelines and shortened questionnaire were sent to the experts during the second round.

**Table 2.** Theme-based revisions to CBT guidelines.

Theme-based revisions to CBT guidelines	
Theme	Revision implemented
1. The CBT-informed guidelines are clear and concise	• Added ‘Key Takeaway’ summaries after each section, condensing the main principles into 1–2 bullet points. • Created a quick-reference infographic that summarises the main cognitive, behavioural and emotional techniques. (e.g., ‘If the patient struggles with overwhelming emotions, see emotional regulation techniques’). • Simplified language and structure
2. There is a need for information regarding role clarification	• Identified the HCPs that may benefit from employing the guidelines (doctors, nurses, dieticians, health educators) and their respective roles. • Listed the responsibilities of the HCPs, such as providing psychoeducation, facilitating cognitive restructuring, and assigning homework. • Listed the patient responsibilities, which could include self-monitoring, completing between-session activities, and reporting challenges and setbacks.
3. It is important to consider the patient’s readiness to change before and during the implementation of the guidelines	• Integrated Prochaska’s Transtheoretical Model as a readiness screening tool linking thoughts and behaviours to the readiness stages • Goal setting highlighted following the SMART guideline and the collaborative nature. • Proposed scaling question to monitor thoughts, behaviours, emotions or patient-identified areas
4. The healthcare practitioners anticipate the guidelines to be time-consuming.	• Developed worksheets that can be used by the HCPs or the patient, which included: • An infographic of the commonly encountered thoughts, emotions and behaviours (HCP). Assisting the HCPs in identifying these components • A psychoeducation worksheet titled, what is CBT? • A thought record worksheet

The panel’s feedback during the second round praised the content’s comprehensiveness and practicality, echoing the first round’s feedback and reaffirming its significance for HCPs. Most experts commended specific improvements, such as including key takeaway messages at the end of each section, integrating Prochaska’s stages of change to assess patient readiness, clarifying roles, and adding worksheets for HCPs and patients (Prochaska et al., 2015). These changes were seen as valuable enhancements to the guidelines. Some panel members suggested minor technical adjustments to spelling and grammar, and a few experts proposed adding additional CBT-related components. Basic CBT skills and techniques were included to ensure simplicity for HCPs unfamiliar with CBT. Overall, this feedback played a crucial role in refining the guidelines, making them adaptable and accessible for HCPs, diverse populations, and various clinical settings.

## Discussion

The study aimed to evaluate the content, quality, and structure of the CBT-informed guidelines Upon their first round of evaluation, the Delphi panel provided feedback that was summarised into four themes, namely (1) the CBT-informed guidelines are clear and concise, (2) there is a need for information regarding role clarification, (3) it is important to consider the patient’s readiness to change before and during the implementation of the guidelines and (4) the healthcare practitioners anticipate the guidelines to be time-consuming. The second-round analysis found that the changes made enhanced the guidelines’ efficiency and feasibility. Minor technical changes were proposed, and there was a general improvement in the guidelines’ comfort and ease of implementation. The guidelines were divided into three phases: the introduction phase, the working phase, and the consolidation phase. The introduction phase aimed to familiarise patients with the CBT model by first assessing their readiness to change, providing psychoeducation, setting collaborative goals, and implementing a mood check. The working phase focussed on the emotional, behavioural, and cognitive elements that required change. The consolidation phase aimed to solidify the skills and techniques acquired by the patient by setting homework, identifying resources, and setting an action plan for future challenges. During each phase, the CBT guidelines aimed to educate patients, promote cognitive flexibility (including cognitive awareness, reappraisal, and acceptance), and assign homework tasks. Similarly, research findings reported that CBT interventions are effective when emphasising components such as psychoeducation, cognitive restructuring, and setting homework assignments (Andreae et al., 2020; Li et al., 2023). Accordingly, Abbas et al. (2023) and Motevalli et al. (2023) found that these strategies facilitate cognitive and emotional functions related to effective self-management. The CBT guidelines were based on core CBT principles but were not intended as a therapeutic intervention, and their format and delivery method were adapted accordingly. The phases served as guidelines for HCPs to determine which techniques and skills from each phase should be introduced and practised by the patient. This format aims to make the guidelines more feasible, cost-effective, and accessible than traditional CBT interventions. An infographic summarising the phases and their respective skills and techniques was provided to the experts. It aimed to orient experts and enable HCPs to easily navigate to their patients’ required skills, techniques, or regulatory strategies. The second round’s feedback reverberated the guidelines’ clarity and practicality. The experts stated that the changes succinctly articulated the guidelines’ goal while remaining comprehensive enough to ensure thorough implementation and the continuation of the intervention. Guo et al. (2022) adapted their programme and reported positive effects on diabetes distress, emotion regulation, and self-management. Similarly, Jenkinson et al. (2022), Li et al. (2023), and Visagie et al. (2023) for peer review reported that the presence of core CBT components contributed to the efficacy of an intervention. An additional aspect requiring attention was clarifying the roles and responsibilities of the HCP and the patient. This was addressed following the first round of feedback, and the guidelines were amended to specifically state the aims for HCPs, imploring HCPs to do a needs analysis of their patient and orienting them within each phase. The fundamental role of an HCP delivering the guidelines is to foster the patient’s self-efficacy by placing them at the centre of self-management. The guidelines aimed to facilitate this process by assessing patient readiness, fostering collaboration through goal setting and resource identification, and enabling patient responsibility through homework assignments and action plans. Research has found that patient-centred interventions promoted effective self-management outcomes (AlHaqwi et al., 2023; Asmat et al., 2022). Kunkes et al. (2022) reported that this approach allowed HCPs to understand their patients’ values and priorities better, providing a clearer rationale behind self-management decisions. Similarly, a systematic review by Asmat et al. (2022) highlighted the need to explore and understand the situational and sociocultural factors that affect self-management and to consider these factors when implementing patient-centred care. In accordance, the guidelines aimed to present a framework that could be tailored to the individual’s needs, financial resources, and personal beliefs, making them more relatable. Role clarification extended to which HCPs can implement these guidelines and how much time the implementation will require. Owing to the guidelines’ non-therapeutic nature, they aim for various HCPs to implement these skills and techniques. Currently, few psychologists, psychiatrists, and multidisciplinary teams are based at the clinics that serve individuals with type 2 diabetes, especially in developing countries (Guo et al., 2022). Thus, having multiple HCPs who can provide CBT skills and techniques can enhance accessibility. Visagie et al. (2023) conducted a review of CBT interventions for type 2 diabetes self-management and found that several HCPs were pre-trained in various CBT formats. Interventions were still effective, and having various HCPs trained to implement programmes promoted accessibility, community engagement, and resource availability (Visagie et al., 2023). Similarly, Guo et al. (2022) emphasised the importance of training HCPs to implement basic mental health interventions to address the limitations of human-intensive, expert-based interventions and to increase participants’ engagement. Currently, the guidelines require no formal training. The current scope of the research was to develop the guidelines as a non-therapeutic approach, and future research is planned to address and develop training programmes for the CBT guidelines. No time indications or limits were proposed for the different phases to promote engagement and ease adherence to the guidelines, and additional resources and changes were developed in response to the first-round feedback. These resources and changes were designed to orient HCPs to the most valuable takeaway message and to provide tangible examples that facilitate their proficiency and autonomy in implementing the guidelines. During the second round, specific feedback acknowledged these changes and noted that the key takeaway messages, worksheets, and clearly defined roles clarified the previously expressed confusion about roles.

### Strengths and limitations

This study provides insights into the key CBT skills and techniques that HCPs can use to facilitate self-management in adults with T2DM. Ultimately, it highlights the critical role of HCPs in their patients’ diabetes care. The diverse and qualified expert panel ensured the reliability of the Delphi results. This study, however, is not without limitations. The recruited experts represented diversity in expertise, age, and years of experience. However, there was a lack of ethnic diversity. Furthermore, although the panel captured the core frontline healthcare providers in South African T2DM management, it did not include other healthcare provider groups that may contribute to diabetes care. This focussed composition was deliberate to ensure the guidelines’ feasibility for the most common care pathway, with future adaptation for other professional groups remaining an important research direction. In addition, a trial questionnaire could have been advantageous for ensuring that the panel effectively managed its design, length, and complexity. Future studies could include a focus group employing the Delphi method to enhance triangulation. A longitudinal approach to the Delphi method can be adopted by revisiting and incorporating feedback at different time points to assess how outcomes have changed. Furthermore, there is a need for continued research to develop training opportunities for HCPs to implement CBT guidelines. The versatile utilisation of the guidelines can be explored by researching different formats, such as a digital intervention or a group format, or by developing the guidelines into a therapeutic intervention.

## Conclusion

These findings highlight the challenges and multifaceted nature of T2DM self-management. The flexible nature of the guidelines addresses the challenges posed and provides a framework for HCPs to adopt a more holistic approach to self-management. In resource-limited settings, time constraints and other logistical challenges may hinder the implementation of full-fledged CBT programmes. A more relaxed framework can aid HCPs in engaging more extensively with individuals with type 2 diabetes, thereby prioritising the most relevant and impactful CBT components and making integration into existing healthcare systems more feasible.

## Data Availability

The data supporting this study’s findings are not publicly available due to ethical and confidentiality restrictions, as the qualitative data could identify participants within a small, specialised expert panel. De-identified excerpts relevant to the findings are included within the article. Further data may be made available from the corresponding author upon reasonable request and with permission from the North-West University Health Research Ethics Committee.
